# Biochemical Association of MTHFR C677T Polymorphism with Myocardial Infarction in the Presence of Diabetes Mellitus as a Risk Factor

**DOI:** 10.3390/metabo13020251

**Published:** 2023-02-09

**Authors:** Tauqeer Hussain Mallhi, Momina Shahid, Kanwal Rehman, Yusra Habib Khan, Abdullah Salah Alanazi, Nasser Hadal Alotaibi, Muhammad Sajid Hamid Akash, Muhammad Hammad Butt

**Affiliations:** 1 Department of Clinical Pharmacy, College of Pharmacy, Jouf University, Sakaka 72388, Saudi Arabia; 2 Department of Pharmacy, The University of Faisalabad, Faisalabad 38000, Pakistan; 3 Department of Pharmaceutical Chemistry, Government College University, Faisalabad 38000, Pakistan; 4 Department of Pharmacy, The Women University, Multan 60000, Pakistan; 5 Department of Medicinal Chemistry, Faculty of Pharmacy, Uppsala University, 75123 Uppsala, Sweden

**Keywords:** MTHFR gene polymorphism, myocardial infarction, myocardial infarction with diabetes mellitus, homocysteinemia, tetra-ARMS PCR

## Abstract

Myocardial infarction (MI) is a cardiovascular disease that occurs due to the blockage of the coronary artery. Subsequently, cardiac muscles receive a lower oxygen supply, which leads to the death of cardiac muscles. The etiology of MI is linked to various environmental, occupational, and genetic factors. Various studies have been conducted on the polymorphism of genes involved in MI. Previous studies have shown that different variants of the methylene tetrahydrofolate reductase (MTHFR) gene are involved in causing MI by altering the metabolism of folate and homocysteine. However, the genetic polymorphism of MTHFR C677T (rs1801133) and its association with MI in the presence of diabetes mellitus (DM) as a risk factor still needs to be investigated. This study recruited 300 participants who were divided into three groups, i.e., the control, MI, and MI-DM. The blood samples collected from the study participants were subjected to various biochemical tests and their clinical parameters were monitored. MTHFR C677T (rs1801133) genotyping was performed by Tetra ARMS PCR using predetermined primers. The MTHFR C677T (rs1801133) polymorphism was associated with MI in the presence of DM as a risk factor among the participants. The MTHFR C677T (rs1801133) T/T homozygous genotype was found to be significant among MI patients in the presence of DM as a risk factor.

## 1. Introduction

Myocardial infarction (MI) is a cardiovascular disease and the main cause of death globally [1]. It is a significant clinical issue because of its mortality [2]. It occurs by the total blockage of the coronary artery, which stops oxygen and nutrients from reaching the heart and causes cell death. MI either happens suddenly or in a devastating way that could cause hemodynamic valve deterioration and mortality [3]. Atherosclerosis leads to coronary artery disease (CAD), which is characterized by the constriction of coronary vessels that occurs after the development of atherosclerotic plaques. Ultimately, endothelial attrition and subsequent thrombosis and plaque rupture induce MI [4]. Cytokines and enzymes released by inflammatory cells weaken the plaque, break down the plaque and lead to the production of thrombus, which causes CAD and acute coronary syndrome [5]. Immune cells such as neutrophils, monocytes, macrophages, mast cells, T cells, and dendritic cells also play a significant role in directing this pathological situation [6]. CD4 T cells, CD8 T cells, B-lymphocytes, and macrophages found within the mononuclear cell infiltrates are primarily responsible for cardiomyocyte destruction [7]. CD4 T cells and their subsets activate and follow complex patterns to coordinate tissue clearance, scar formation, and immune resolution post-MI [8]. T cells prevent the production of pro-inflammatory factors and lower the apoptosis of cardiomyocytes [9]. A series of quick early innate immune responses are activated by MI and are followed by the activation of the adaptive immune system. The activation of immune responses is protective and helps to recover, creates scars, and re-establishes homeostasis [10]. A lack of CD4 T cells worsens myocardial damage and increases the chance of death [11].

MI is a complicated, multifactorial, and polygenic cardiovascular disease that results from the interplay between inherited predisposition and ecological factors [12]. Conventional risk aspects, such as nutrition, adiposity, insulin resistance, high blood pressure, hyperlipidemia, inflammatory processes, tobacco smoking, and excessive alcohol intake, have been shown to predict about 50% of an individual patient’s risk of having a cardiovascular incident, with the remainder due to genetic variables [13,14,15]. The genetic vulnerability may result from alteration and mutations in a number of genes, primarily those implicated in the clotting of blood, maintenance of blood pressure and metabolism of lipid, glucose, and homocysteine [13]. DM increases the incidence of MI in individuals and is a risk factor for the progression of CAD [16]. Progression of atherosclerosis in DM is due to numerous factors, such as DM, oxidative stress, advanced glycation end product, cholesterol, autonomic dysfunction, metabolic abnormalities, excessive inflammatory cytokines, and hereditary factors [17]. Neutrophils, regulatory T cells, myeloid-derived suppressor cells, activated dendritic cells, central memory CD4, and CD8 T cells lead to immune cell infiltration and development of cardiomyopathy in diabetic patients [18].

There is strong evidence that a higher level of total plasma homocysteine is a major health concern for vascular diseases, such as CAD and MI. The plasma concentrations of homocysteine can be increased due to a variety of hereditary or dietary conditions [19]. The methylenetetrahydrofolate reductase (MTHFR) gene has drawn considerable attention as it has been linked to an increased plasma level of homocysteine, which has been associated with an increased risk of MI [20]. Homocysteine, an amino acid that contains sulfur, has significance in CAD and is an absolute atherosclerotic risk of coronary and peripheral vascular lesions [5].

**Figure 1:**
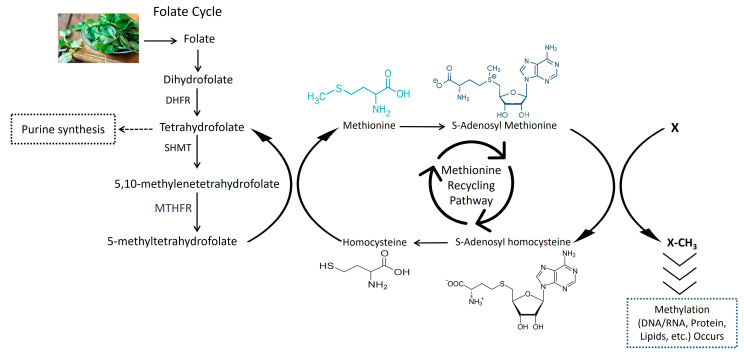
Mechanism of methylene tetrahydrofolate reductase (MTHFR) in folate and homocysteine metabolism.

MTHFR is an important enzyme that plays a significant role in the metabolism of homocysteine. The transformation of homocysteine to methionine requires MTHFR, which catalyzes the reaction between 5,10-methylenetetrahydrofolate and 5-methyltetrahydrofolate [21], as shown in Figure 1. The MTHFR gene has 11 exons, a length of 2.2 kb, and is found on chromosome 1p36.3 [22]. The dbSNP database reports that the MTHFR gene has at least 247 single nucleotide polymorphisms (SNPs). The most common polymorphisms of MTHFR gene’s DNA sequence variants are MTHFR C677T (rs1801133) and MTHFR A1298C (rs1801131) SNPs [22,23]. The C677T (rs1801133) polymorphism involves the C to T transformation of the MTHFR gene coding sequence at 677 nucleotides, resulting in the substitution of ala-222 with valine, and the A1298C (rs1801131) polymorphism involves the A to C transformation of the MTHFR gene at 1298 nucleotides, resulting in the substitution of Glu-429 with alanine. These are the genetic factors of increased homocysteine levels and thrombotic disposition [24]. Several studies have also demonstrated that elevated homocysteine levels increase the chances of developing cardiovascular diseases [25,26].

The association between the polymorphism of MTHFR C677T (rs1801133) and MI in the presence of DM as a risk factor has not yet been investigated among the Pakistani population. In this study, we have investigated the association of MTHFR C677T (rs1801133) genotype with MI among the Pakistani population and the association of MTHFR C677T (rs1801133) gene polymorphism with MI in the presence of DM, being a risk factor, and we have compared this with healthy individuals.

## 2. Materials and Methods

### 2.1. Ethical Considerations

This study was approved by the Ethical Review Committee, Government College University, Faisalabad, Pakistan (Ref. No. GCUF/ERE/36). Informed written consent was obtained from all participants before collecting blood samples.

### 2.2. Study Design

The current study was a case–control study that was conducted to determine the relationship between the frequencies of MTHFR C677T (rs1801133) gene mutation in MI patients in the presence of DM as a risk factor by analyzing the polymorphism using Tetra-ARMS PCR. The sampling was performed in the Allied Hospital, Faisalabad and the Faisalabad institute of Cardiology, Faisalabad, Pakistan, according to the guidelines approved by the ethical committee of Government College University, Faisalabad, Pakistan. We recruited a total of 300 study participants who were divided into three groups: Group I included the control patients (*n* = 100), group II included MI patients (*n* = 100), and group III (*n* = 100) included MI with diabetic patients (MI-DM). The study participants were briefly informed of the purpose of the research and written consent was obtained from all the participants before collecting blood. The blood specimen was collected from the target population and underwent various biochemical analyses and Tetra-ARMS PCR for genotyping to observe the genetic polymorphism.

### 2.3. Standards for Inclusion and Exclusion of Study Participants

Study participants of age 30–70 were selected. No participants below the age of 30 or older than 70 were recruited in this study. Study participants who had any other medical history, history of alcohol intake, or any other disorder and used medication for any comorbidity for a long period were excluded from this study. Standard measurements of cardiac biomarkers and electrocardiogram (ECG) were used for the diagnosis of MI and MI-DM patients. MI-DM patients recruited for this study had an HbA1c value greater than 5.7%. Similarly, MI Patients were receiving antiplatelet (aspirin and clopidogrel) and anticoagulant therapy (heparin and streptokinase), whereas patients with MI-DM were receiving antidiabetic (insulin, glipizide, and metformin), antiplatelet (aspirin and clopidogrel), and anticoagulant (heparin and streptokinase) therapies.

### 2.4. Measurement of Clinical Parameters

Vital signs such as pulse, blood pressure, respiration rate, and temperature were assessed at the time of sample collection. A traditional mercury sphygmomanometer was used to measure the systolic and diastolic pressures (mmHg). A routine ECG was also performed and the patients with ECG abnormalities were noted as a diseased group. The ECG tracings of the control group were normal and there were no clinical signs of MI in the control group.

### 2.5. Collection of Blood Specimen

Blood was taken from the cubital vein of each subject in two different tubes (EDTA and gel clot activator vacutainer). About 3 mL of blood was taken for biochemical analysis in a gel clot activator vacutainer, while about 4 mL blood was taken for DNA extraction in an EDTA tube. The blood specimen was transported from the hospital to the laboratory in an ice box or ice pack. The gel clot activator vacutainer was then centrifuged to separate the blood serum and stored at −20 °C for biochemical evaluation. However, for the extraction of DNA, blood specimens in EDTA tubes were used.

### 2.6. Analysis of Biochemical Parameters

The separated serum from the blood specimen of study participants was used for the assessment of various biochemical parameters, i.e., glycemic profile (random blood sugar (RBS) and glycated hemoglobin A1c (HbA1c)), cardiac biomarkers (troponin i, creatine kinase-MB (CK-MB), lactate dehydrogenase (LDH), and creatinine phosphokinase (CPK)), liver enzymes (alanine transaminase (ALT) and aspartate aminotransferase (AST)), renal profile (urea and creatinine), lipid profile (triglyceride), homocysteine levels, and folate levels, which are involved in MI and DM, by the use of their respective assay kits with the help of a biochemical analyzer (Microlab-300 BIOBASE, China).

### 2.7. Extraction of DNA

Genomic DNA was extracted manually from the obtained blood specimens. Blood was collected in Eppendorf tubes and RBC lysis buffer was added to them. The sample was shaken and then centrifuged for 2 min at 7000 rpm. The pellet was broken with the help of a vortex mixer and rinsed well with RBC lysis buffer. Then, nucleic acid lysis buffer was added, followed by saturated NaCl (5 M) and chloroform into the Eppendorf tube. Then, the sample was again mixed and centrifuged for 2 min at 7000 rpm and the supernatant was transferred to a new Eppendorf tube and cold ethanol was added. The sample was shaken and centrifuged for 1 min at 12,000 rpm and then the supernatant was discarded. At the end, TE buffer was added and the mixture was vortexed. Finally, the sample was kept in the Eppendorf tube at −20 °C until further analysis for genotyping.

### 2.8. Purification and Estimation of DNA

The DNA concentration was determined after DNA extraction. For quantitative analysis, the nano-drop method was used, while for the qualitative analysis, the gel electrophoresis technique was used. The purity of the extracted DNA was examined by placing the samples on 2% agarose gel and an absorbance ratio of 260/280 was used to calculate the number of nucleic acids.

### 2.9. Genotyping of MTHFR Gene (rs1801133)

To genotype the SNP of the MTHFR C677T (rs1801133) gene, Tetra-ARMS PCR was used. Three types of genotypes (C/C, C/T, and T/T) were found for the MTHFR C677T (rs1801133) gene. The base pair size used for Tetra ARMS PCR was 86 bp and 146 bp. For the amplification of the MTHFR C677T (rs1801133) gene, two forward primers and two reverse primers were used (Table 1). At the start of the reaction, two non-allele specific primers were amplified by the outer primers which produced outer fragments that served as a template for the attachment and elongation of inner primers.

A Thermocycler Master Cycler Gradient was used to perform PCR. PCR was carried out in a 20 µL container with 1 µL of DNA sample, 10 µL Master Mix, 0.1–0.5 mM of each primer, and 8 µL of DD-H_2_O. The initial denaturation temperature was 95 °C for 5 min, further followed by 40 cycles of denaturation at 94 °C for 30 s. Annealing was performed at 52 °C for 75 s, followed by extension at 72 °C for 40 s. The final extension temperature was again 72 °C for about 7 min. After the completion of the PCR reaction, 20 μL of PCR resultant product was placed in the well with 2% agarose gel, dyed with ethidium bromide, soaked in TAE buffer, and let to run in an electric field. The gel was then examined under UV light using a gel documentation system (InGenius3, Syngene, UK).

### 2.10. Statistical Analysis

The data were represented as means ± SD and statistical analysis was performed using GraphPad Prism 5 (version 5.01). Statistically significant differences among the study groups were set at *p* < 0.05 and analyzed by using one way ANOVA and Tukey’s Multiple Comparison test. The significant difference in genotype frequency of MTHFR C677T (rs1801133) among the study groups was calculated by using Fisher’s exact test.

## 3. Results

### 3.1. Clinical Profile and Anthropomorphic Characteristics

The mean systolic blood pressure was 114.2 ± 12.41 mmHg for the controls. The mean systolic blood pressure was 146.4 ± 22.72 mmHg for the MI study group and 174.6 ± 48.46 mmHg for the MI-DM study group. The mean diastolic blood pressure was 74.74 ± 9.88 mmHg for the controls. The mean diastolic blood pressure was 94.6 ± 10.86 mmHg for the MI study group and 108.2 ± 16.29 mmHg for the MI-DM study group. The data were calculated using one way ANOVA and Tukey’s Multiple Comparison test as shown in Table 2. After statistical analysis, it was found that the mean systolic blood pressure was high in the MI-DM study group when compared to the MI study group and the controls. The mean diastolic blood pressure was also high in the MI-DM study group when compared to the MI study group and the controls. The gender distribution shows that the number of females recruited in this study was high in MI and MI-DM study groups when compared to males in all groups. Females are at a high risk of developing MI and MI-DM compared to males. While on the other hand, the smoking status of the study participants showed that there were more smokers in the MI and MI-DM study groups when compared to the non-smokers in these groups. Smokers were at a higher risk of developing MI and MI–DM when compared to non-smokers.

### 3.2. Biochemical Analysis

The glycemic profile (RBS and HbA1c) was significant in MI and MI-DM study groups when compared with that of the controls. The cardiac biomarkers (Troponin i, CK-MB, LDH, and CPK) were found to be significantly high in MI and MI-DM study groups when compared with the controls. The liver enzymes (ALT and AST) were also found to be significantly high in both the MI and MI-DM study groups when compared with the controls. The renal profile (urea and creatinine) and lipid profile (TGs) were found to be significantly high in both the MI and MI-DM study groups when compared with those of the controls. The homocysteine and folate levels were also found to be high in both the MI and MI-DM study groups when compared with the controls. The values of these biochemical parameters are shown in Table 3.

### 3.3. Results of Gel Electrophoresis

**Figure 2:**
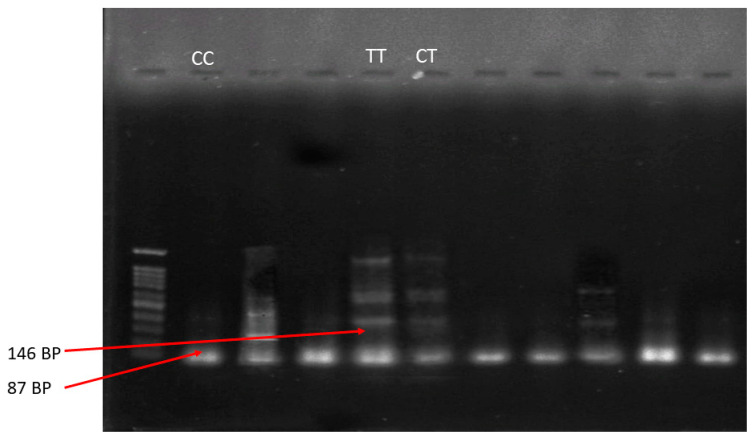
Agarose gel electropherogram of MTHFR (rs1801133), or genotype (CC, TT, and CT) performed by Tetra-ARMS PCR.

The MTHFR C677T (rs1801133) gene polymorphism was amplified and genotyped using Tetra–ARMS PCR method. Three different kinds of MTHFR C677T (rs1801133) genotypes (TT, CT, and CC) were confirmed via PCR results after the confirmation of the gel documentation results. In a single PCR reaction, each sample’s genomic DNA was amplified using the set of two forward (inner and outer) and two reverse (inner and outer) primers. The band size of 87 bp was for the C allele, for the outer primer and the inner primer. Meanwhile, the 146 bp band was for the other inner and outer primer for the T allele. The PCR product was viewed under UV light after gel electrophoresis as shown in Figure 2.

### 3.4. Genotype and Allelic Frequency for the Control, Myocardial Infarction, and Myocardial Infarction with Diabetes Study Group

The genotype (C/C, C/T, and T/T) frequency of MTHFR in the control, MI, and MI-DM study groups showed that the T/T genotype was high among MI and MI-DM study groups when compared to the controls. However, the prevalence of C/C and C/T genotypes was lower among the MI and MI-DM study groups compared to the controls. The allelic distribution of C and T alleles among the study groups showed that the T allele was more prevalent in the MI and MI-DM study groups when compared to the control (Table 4).

### 3.5. Association of SNP among the Control, Myocardial Infarction, and Myocardial Infarction with Diabetes Mellitus among Study Groups

Association of SNP among the control, MI, and MI-DM study groups showed that there was a significant difference for homozygous genotype T/T under the codominance genetic model (OR = 1.74, CI 95% = 0.65–4.63, *p* < 0.05) and overdominance genetic model (OR = 0.50, CI 95% = 0.27–0.91, *p* < 0.05) between the control and MI study group (Table 5). A significant difference was found for homozygous genotype T/T under the codominance genetic model (OR = 0.52, CI 95% = 0.27–0.97, *p* < 0.05) and overdominance genetic model (OR = 0.45, CI 95% = 0.24–0.83, *p* < 0.05) between the control and MI-DM study groups (Table 6). There was also a significant difference for homozygous genotype T/T under the codominance genetic model between the MI and MI-DM study groups (OR = 0.65, CI 95% = 0.46–2.90, *p* < 0.05) (Table 7). SNP association between the control and diseased (MI and MI-DM) groups showed that for the homozygous genotype (T/T), there was a significant difference under the codominance genetic model (OR = 0.53, CI 95% = 0.31–0.90, *p* < 0.05) and overdominance genetic model (OR = 0.47, CI 95% = 0.28–0.79, *p* < 0.05). Hence, this result indicates that the codominant and overdominant T/T genotype might be a risk for the pathogenesis of MI (Table 8).

### 3.6. Clinical and Biochemical Association of MTHFR C677T (rs1801133) SNP

The data for clinical and biochemical parameters are shown in Table 9. The level of systolic blood pressure was high in MI and MI-DM study groups when compared with the control. However, the mean of homozygous genotype T/T was higher in the MI and MI-DM study groups than the C/C and C/T genotypes when compared with control. It was found that blood pressure was elevated in MI and MI-DM study groups when compared with the control, and the mean of homozygous genotype T/T was more than the C/C and C/T genotype. The overall *p*-value was also significant, indicating that the T/T genotype has an association with the pathogenesis of MI.

The level of glycemic profile (RBS and HbA1c) was found to be high in MI and MI-DM study groups when compared with the control, and the mean of homozygous genotype T/T was high in the MI-DM study group compared with the control. The level of cardiac biomarkers (troponin i, CK-MB, LDH, and CPK) was found to be high in MI and MI-DM study groups when compared to the control and the mean of homozygous genotype T/T was high in the MI and MI-DM study groups. The level of liver enzymes (ALT and AST) was found to be significantly increased in MI and MI-DM study groups when compared with the control, and the mean of the homozygous genotype T/T was high in MI and MI-DM study groups. The level of kidney profile (urea and creatinine) was found to be significantly increased in MI and MI-DM study groups when compared to the controls, and the mean of homozygous genotype T/T was high in MI and MI-DM study groups. The level of lipid profile (TGs) was found to be high in MI and MI-DM study groups compared to the control, and the mean of the homozygous genotype T/T was higher in the MI and MI-DM study groups than in the control. The level of homocysteine was found to be high in MI and MI-DM study groups compared to the control, and the mean of the homozygous genotype T/T was high in MI and MI-DM study groups compared to the control. The level of folate was significantly decreased in MI and MI-DM study groups compared to the control. However, the mean of the homozygous genotype T/T was higher in MI and MI-DM study groups when compared to the control (Table 9).

## 4. Discussion

CVD has been identified as a primary cause of death over the years [27]. Numerous findings have suggested that environmental and genetic factors play a critical role in the emergence of CAD [28,29]. MTHFR is considered as an important enzyme for the metabolism of homocysteine and its impairment leads to the develop of CVD. Numerous clinical findings have shown that plasma homocysteine levels lead to CVD and predict the death in patients with CAD independent of other risk factors [30,31]. Various nutritional and genetic factors are known to affect the plasma homocysteine levels [32]. Frosst and co-workers found that the SNP of the MTHFR C677T gene lowers the enzyme’s activity and thermolability. Homozygous mutant TT alleles result in noticeably higher plasma homocysteine levels [33].

Numerous researchers have conducted case–control studies to establish a link between MTHFR mutation and the occurrence of CAD or MI; however, the findings were conflicting due to small sample sizes. A key feature of meta-analysis is that it increases the sample size and statistical power to combine the comparable research to provide a more competitive conclusion [34]. The prevalence of the MTHFR C677T mutation is linked to a reduction in MTHFR activity by 50%, a rise in homocysteine levels, and a fall in folic acid concentration, which worsens endothelial dysfunction and raises the risk of CVD [35].

**Figure 3:**
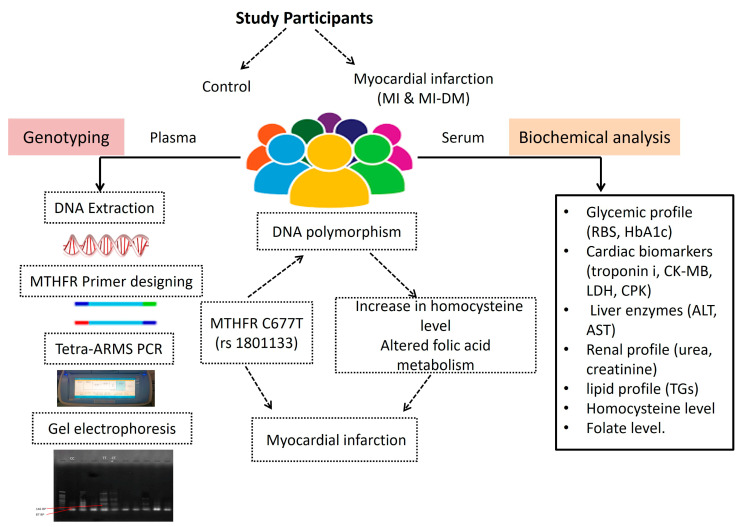
Genotype and biochemical analysis of MTHFR C677T (rs1801133).

A schematic representation of this study is shown in Figure 3. Our data showed that in the MI and MI with diabetes (MI-DM) study groups, there was an increase in homocysteine levels when compared with the controls. Furthermore, the folate level was decreased in MI and MI with diabetes (MI-DM) study groups.

In our study, we analyzed all the diagnostic markers such as cardiac biomarkers (troponin i, CK-MB, LDH, and CPK), liver function test (ALT and AST), renal profile (urea and creatinine), triglycerides, HbA1c, and random blood sugar. There was significant increase in the level of cardiac biomarkers in the MI and MI with diabetes (MI-DM) study groups when compared with the control. Liver enzymes were found to be significantly increased in the MI and MI with diabetes (MI-DM) study groups when compared with the control. The renal profile was significantly increased in MI and MI with diabetic (MI-DM) study groups when compared with the control. There was an increase in triglycerides in the MI and MI with diabetes (MI-DM) study groups when compared with the control. There was significant increase in HbA1c and random blood sugar in the MI and MI with diabetes (MI-DM) study groups when compared with the control.

Furthermore, the genotypic study of MTHFR C677T (rs1801133) showed that the codominant and overdominant homozygous T/T genotype had a high homocysteine level in MI and MI-DM study groups compared to the control and could be the risk factor for the pathogenesis of MI. In harmony with our results, Huh et al. showed that people with the homozygous T/T genotype had a high level of homocysteine, and individuals with the T/T genotype had significantly higher genotype-specific folate threshold levels than those with C/C or C/T genotypes. Interestingly, patients with low folate levels and the TT genotype had 2.2-fold higher probability of coronary artery disease (CAD), while those with high folate levels and the T/T genotype had 1.5-fold decreased probability of CAD [36].

Klerk M. et al. found a relationship between the MTHFR C677T genotype and an increased prevalence of coronary heart disease (CHD) in the presence of low plasma folate levels. These findings showed that elevated homocysteine levels are caused by poor folate metabolism and lead to CHD [37]. Contrary to the above data, Kang et al. discovered that those with the variation of MTHFR have a high chance of developing CAD compared to normal. In that study, only 5% of the controls and 17% of the cardiac patients had thermolabile MTHFR [38]. According to another report, the population of Mexico has a high occurrence of the MTHFR C677T mutation with a high frequency of the T allele. The authors of the same study also noted a significant distribution of the T allele in Korea and Italy. However, a comparatively low incidence of the T allele was discovered in Canada, as well as in North and South Africa [39].

According to Benrahma et al., there is an association between Moroccan patients and the control in terms of the allelic frequency of C677T and the occurrence of cardiovascular disease among the Moroccan population [40]. Movva et al. discovered that the MTHFR C677T variant increased the risk of developing myocardial infarction by four times in the Indian population [41].

Eftychiou et al. found no association between MTHFR polymorphism (C677T and A1298C) in patients and the control. The mutant homozygous 677T/T was present more in patients than in the control, while mutant homozygous 1298CC was also present more in patients than in the control [42]. Allawi et al. found that the MTHFR C677T C/T and T/T alleles were present in the Iraqi population and that the MTHFR C677T (T/T) genotype was a risk for developing myocardial infarction [43].

According to Alizadeh et al., the MTHFR C677T mutation was linked to an increased risk of MI in Africa and North America [2]. According to Chen et al., there was a significant interaction between the MTHFR C677T polymorphism and IHD risk. The MTHFR C677T homozygous (T/T) genotypes were highest in the Hispanic, East Asian, African, Caucasian, Middle Eastern, and South Asian populations [44].

Isordia-Salas et al. showed that the mutation of the MTHFR C677T T/T genotype did not increase the incidence of myocardial infarction in the Mexican population [15]. Patti et al. found that the prevalence of myocardial infarction with MTHFR C677T was high with T/T genotype when compared to those with C/T and C/C genotypes, and the T/T genotype independently was a risk factor for myocardial infarction with a specificity of 90%. The greatest homocysteine levels were found in myocardial infarction and the T/T genotype. The T/T homozygosity is independently linked to myocardial infarction [21].

Isordia-Salas et al. found that the T allele of the MTHFR C677T polymorphism is a risk for myocardial infarction in the adults of Mestizos in Mexico [45]. Some of the important studies are mentioned in Table 10.

In conclusion to our results, this study is the first report on the association between the MTHFR C677T (rs1801133) polymorphism and MI patients and MI-DM patients among the Pakistani population. Our data showed a significant difference in the occurrence of the homozygous genotype T/T mutation of the MTHFR C677T (rs1801133) gene among MI patients and MI-DM patients and the control, which suggests an increased risk of MI among the Pakistani population. This study also aimed to individualize the treatments by promoting the rational and direct use of medication, hence removing clinical trial and error. It will reduce the morbidity and mortality rate and will enhance the patient benefits as well as reduce the cost of treatment. This pharmacogenomics study will also help to discover new drug targets and also reduce adverse effects. For future health, it enables the prediction of individual’s health risk, the development of personalized medical treatment, and leads to the prevention of disease through drug design and development. Finally, we also state some and/or certain limitations of this study, including the small sample size, limited population, classification of participants into groups, and short time duration.

## 5. Conclusions

Our study indicated a high association of MI with the MTHFR polymorphism in the presence of diabetes as a risk factor. However, the predisposition to MI might be due to genetic mutation. We suggest that numerous genome-wide association studies (GWAS) and epidemiological studies must be performed to further illuminate the pathogenesis of MI. An analysis of the relationship between the disease and genetic variation can help in rationalizing the course of treatment and lowering the medication errors. Along with better health outcomes, it can also lower the treatment costs and improve quality of life. The pathophysiology of the disease can be better understood with the use of a pharmacogenomics approach, which may ultimately result in the development of novel and target-specific medications.

## Figures and Tables

**Table 1:** The Primer Sequence of MTHFR Variant C677T (rs1801133).

Gene Primers	Primer Sequence
Forward inner primer	5′GAAGGAGAAGGTGTCTGCGGGAAT3′
Forward outer primer	5′CCGAAGCAGGGAGCTTTGAGG3′
Reverse inner primer	5′CCCTCACCTGGATGGGAAAGAT3′
Reverse outer primer	5′AGCAAAGCTGCGTGATGATGAAATAGG3′

**Table 2:** Comparison of clinical parameters with anthropomorphic parameters among the control, myocardial infarction (MI), and myocardial infarction with diabetes mellitus (MI-DM) study groups.

Characteristics	Control	MI	MI-DM	*p*-Value
No. of participants	100	100	100	
**Age (Years)**				
Age	46.94 ± 8.71	52.2 ± 9.484	52.26 ± 9.76	<0.0001
**Blood pressure (mmHg)**				
Systolic Blood Pressure	114.2 ±12.41	146.4 ± 22.72	174.6 ± 48.46	<0.0001
Diastolic Blood Pressure	74.74 ± 9.88	94.6 ± 10.86	108.2 ± 16.29	<0.0001
**Gender wise distribution (sex)**				
Males	30	33	49	<0.0001
Female	70	67	51	<0.0001
**Smoking status**				
Smoker	12	67	75	<0.0001
Non smoker	88	33	25	<0.0001
**Physical activity**				
Bad	14	56	51	<0.0001
Good	86	44	49	<0.0001

The *p*-value was calculated by GraphPad prism (version 5.01) using one-way ANOVA and Tukey’s Multiple Comparison test.

**Table 3:** Biochemical analysis of the control, myocardial infarction (MI), and myocardial infarction with diabetes mellitus (MI-DM) study groups.

Biochemical Test	Control (*n* = 100)	MI (*n* = 100)	MI-DM (*n* = 100)	*p*-Value
Random blood sugar	94.24 ± 11.09	245.2 ± 119.5	304.1 ± 108.7	<0.05
Hemoglobin A1C	4.724 ± 0.4901	5.464 ± 1.151	7.892 ± 0.6675	<0.05
Troponin i	0.202± 0.770	2.610 ± 1.371	3.637 ± 1.323	<0.05
Creatinine kinase MB	21.96 ± 2.238	42.10 ± 14.75	44.82 ± 18.55	<0.05
Lactate dehydrogenase	372.0 ± 50.69	482.6 ± 100.9	518.4 ± 53.48	<0.05
Creatinine phosphokinase	128.1 ± 35.77	261.6± 91.96	287.6± 87.81	<0.05
Alanine transaminase	18.78 ± 6.137	36.12 ± 7.299	45.58 ± 8.924	<0.05
Aspartate aminotransferase	26.10 ± 9.745	38.42 ± 13.99	51.76 ± 11.05	<0.05
Urea	30.72 ± 6.523	40.42 ± 11.65	49.28 ± 25.28	<0.05
Creatinine	0.8400 ± 0.147	1.846 ± 0.6193	2.208 ± 0.954	<0.05
Triglycerides	90.76 ± 21.98	157.5 ± 4.945	164.6 ± 10.80	<0.05
Homocysteine	8.200 ± 2.636	49.80 ± 14.18	81.80 ± 13.99	<0.05
Folate	29.64 ± 6.928	8.240 ± 2.760	4.240 ± 1.615	<0.05

The *p*-value was calculated by GraphPad prism (version 5.01) by using one-way ANOVA and Tukey’s Multiple Comparison test.

**Table 4:** Genotype and allelic frequency of MTHFR C677T (rs1801133) gene polymorphism in the control, myocardial infarction (MI), and myocardial infarction with diabetes mellitus (MI-DM) study groups.

Groups	Allele		Genotype			*p*-Value
	T	C	C/C	C/T	T/T	--
Control	141	59	71	270	2	0.0001
MI	147	53	53	25	22	
MI-DM	146	54	59	23	18	

The *p*-value was calculated by SNP Stat software (http://bioinfo.iconcologia.net/SNPstats) (accessed on 1 December 2022) by using Fisher’s exact test and Hardy-Weinberg equilibrium analysis.

**Table 5:** Association of MTHFR C677T (rs1801133) single nucleotide polymorphism in the control and myocardial infarction (MI) study groups (*n* = 200).

Genetic Model	Genotype	Control (*n* = 100)	MI (*n* = 100)	OR (95% CI)	*p*-Value
Codominance	C/C	53(53%)	61 (61%)	1.00	0.04
	C/T	40(40%)	25 (25%)	0.54 (0.29–1.01)	
	T/T	7 (7%)	14 (14%)	1.74 (0.65–4.63)	
Dominance	C/C	53(53%)	61 (61%)	1.00	0.25
	C/T -T/T	47(47%)	39 (39%)	0.72 (0.41–1.26)	
Recessive	C/T -C/T	93(93%)	86 (86%)	1.00	0.1
	T/T	7 (7%)	14 (14%)	2.16 (0.83–5.61)	
Overdominance	C/C -C/T	60(60%)	75 (75%)	1.0	0.023
	T/T	40(40%)	25 (25%)	0.50 (0.27–0.91)	

The *p*-value was calculated by SNP Stat software by using Fisher’s exact test and Hardy–Weinberg equilibrium analysis.

**Table 6:** Association of MTHFR C677T (rs1801133) single nucleotide polymorphism in the control and myocardial infarction with diabetes mellitus (MI-DM) study groups (*n* = 200).

Genetic Model	Genotype	Control (*n* = 100)	MI-DM (*n* = 100)	OR (95% CI)	*p*-Value
Codominance	C/C	53 (53%)	59 (59%)	1.00	0.006
	C/T	40 (40%)	23 (23%)	2.31 (0.89–5.96)	
	T/T	7 (7%)	18 (18%)	0.52 (0.27–0.97)	
Dominance	C/C	53 (53%)	59 (59%)	1.00	0.39
	C/T-T/T	47 (47%)	41 (41%)	0.78 (0.45–1.37)	
Recessive	C/C-C/T	93 (93%)	82 (82%)	1.00	0.17
	T/T	7(7%)	18 (18%)	2.92 (1.16–7.33)	
Overdominance	C/C-C/T	60(60%)	77 (77%)	1.00	0.009
	T/T	40(40%)	23 (23%)	0.45 (0.24–0.83)	

The *p*-value was calculated by SNP Stat software (http://bioinfo.iconcologia.net/SNPstats) (accessed on 1 December 2022) by using Fisher’s exact test and Hardy–Weinberg equilibrium analysis.

**Table 7:** Association of MTHFR C677T (rs1801133) single nucleotide polymorphism in myocardial infarction (MI) and MI with diabetes mellitus (MI-DM) study groups (*n* = 200).

Genetic Model	Genotype	MI (*n* = 100)	MI-DM (*n* = 100)	OR (95% CI)	*p*-Value
Codominance	C/C	61 (61%)	59 (5%)	1.00	0.04
	C/T	38 (38%)	34 (34%)	0.86 (0.471.57)	
	T/T	10 (10%)	12 (12%)	0.65 (0.46–2.90)	
Dominance	C/C	61 (61%)	59 (59%)	1.00	0.77
	C/T-T/T	39 (39%)	41 (41%)	1.92 (0.62–1.91)	
Recessive	C/C-C/T	86 (86%)	82 (82%)	1.00	0.44
	T/T	14 (14%)	18 (18%)	1.35 (0.63–2.89)	
Overdominance	C/C-C/T	75(75%)	77 (77%)	1.00	0.74
	T/T	25 (25%)	23 (23%)	0.90 (0.47–1.50)	

The *p*-value was calculated by SNP Stat software (http://bioinfo.iconcologia.net/SNPstats) (accessed on 1 December 2022) by using Fisher’s exact test and Hardy–Weinberg equilibrium analysis.

**Table 8:** Association of MTHFR C677T (rs1801133) single nucleotide polymorphism in control and myocardial infarction (MI) with and without diabetes mellitus (DM) study groups (*n* = 300).

Genetic Model	Genotype	Control (*n* = 100)	MI with and without DM (*n* = 200)	OR (95% CI)	*p*-Value
Codominance	C/C	53 (53%)	120 (60%)	1.00	0.004
	C/T	40 (40%)	48 (24%)	2.02 (0.84–4.86)	
	T/T	7 (7%)	32 (16%)	0.53 (0.31–0.90)	
Dominance	C/C	53 (53%)	120 (60%)	1.00	0.25
	C/T-T/T	47 (47%)	80 (40%)	0.89 (0.55–1.43)	
Recessive	C/C-C/T	93 (93%)	168 (84%)	1.00	0.096
	T/T	7 (7%)	32 (16%)	2.53 (1.07–5.96)	
Overdominance	C/C-C/T	60(54%)	152 (76%)	1.00	0.0046
	T/T	40 (46%)	48 (24%)	0.47 (0.28–0.79)	

The *p*-value was calculated by SNP Stat software (http://bioinfo.iconcologia.net/SNPstats) (accessed on 1 December 2022) by using Fisher’s exact test and Hardy–Weinberg equilibrium analysis.

**Table 9:** Clinical and biochemical parameters for control, myocardial infarction (MI), and myocardial infarction with diabetes mellitus (MI-DM) according to genotype (C/C, C/T, and T/T) of MTHFR C677T (rs1801133). The data are presented as means ± SD.

Parameters	Control (*n* = 100)			MI (*n* = 100)			MI-DM (*n* = 100)			*p*-Value
	C/C	C/T	T/T	C/C	C/T	T/T	C/C	C/T	T/T	
**Clinical**										
Systolic BP	114.2 ± 12.47	113.5 ± 12.33	122.5 ± 12.58	144.0 ± 22.51	147.6 ± 25.41	146.0 ± 11.74	176.3 ± 49.80	166.5 ± 44.71	190.0 ± 52.05	<0.05
Diastolic BP	74.40 ± 9.930	73.48 ± 9.711	85.00 ± 5.774	94.23 ± 10.91	94.74 ± 11.56	96.00 ± 8.433	110.0 ± 17.80	104.7 ± 14.82	110.0 ± 12.06	<0.05
**Biochemical**										
RBS	94.06 ± 11.11	95.04 ± 11.41	87.50 ± 4.509	241.0 ± 119.6	238.1 ± 117.7	294.1 ± 126.4	303.4 ± 115.8	292.4 ± 103.0	340.7 ± 89.43	<0.05
HbA1c	4.724 ± 0.492	4.743 ± 0.5027	4.500 ± 0.316	5.431 ± 1.152	5.687 ± 1.228	4.790 ± 0.354	7.863 ± 0.682	7.838 ± 0.655	8.175 ± 0.613	<0.05
Troponin i	0.0224 ± 0.0118	0.0230 ± 0.011	0.0125 ± 0.005	2.570 ± 1.367	2.728 ± 1.435	2.373 ± 1.221	3.598 ± 1.332	3.526 ± 1.329	4.128 ± 1.258	<0.05
CK-MB	21.96 ± 2.249	22.17 ± 2.122	19.50 ± 2.517	41.85 ± 14.71	41.26 ± 14.19	46.60 ± 17.69	44.02 ± 18.40	44.82 ± 18.47	48.42 ± 20.60	<0.05
LDH	372.0 ± 50.95	375.2 ± 51.54	335.0 ± 23.80	487.1 ± 103.1	477.6 ± 100.6	478.5 ± 99.15	516.4 ± 52.39	515.4 ± 48.48	535.8 ± 71.28	<0.05
CPK	128.5 ± 36.23	130.4 ± 34.16	96.25 ± 43.47	259.8 ± 91.66	264.9 ± 99.86	257.9 ± 65.86	293.7 ± 90.14	281.6 ± 89.79	276.7 ± 75.12	<0.05
ALT	18.78 ± 6.169	18.72 ± 5.935	19.50 ± 9.609	35.48 ± 7.932	36.74 ± 7.028	37.10 ± 4.630	44.26 ± 9.961	47.09 ± 8.083	47.23 ± 5.246	<0.05
AST	26.31 ± 9.786	25.72 ± 9.960	28.00 ± 8.524	38.13 ± 13.86	37.97 ± 13.72	41.60 ± 16.65	51.46 ± 10.75	50.68 ± 12.28	56.17 ± 8.032	<0.05
Urea	30.72 ± 6.556	30.74 ± 6.794	30.50 ± 3.109	40.23 ± 11.52	41.13 ± 12.56	38.70 ± 9.238	49.33 ± 26.19	52.65 ± 27.20	39.50 ± 9.463	<0.05
Creatinine	0.840 ± 0.1485	0.991 ± 0.055	1.000 ± 0.01	1.817 ± 0.627	1.832 ± 0.620	2.050 ± 0.594	2.193 ± 0.953	2.286 ± 1.058	2.058 ± 0.644	<0.05
Triglycerides	90.76 ± 22.09	91.17 ± 22.88	86.00 ± 9.201	157.4 ± 4.956	157.2 ± 4.600	159.4 ± 6.204	164.6 ± 10.64	164.6 ± 11.65	164.6 ± 9.867	<0.05
Homocysteine	8.200 ± 2.650	8.065 ± 2.703	9.750 ± 1.258	49.33 ± 14.18	49.47 ± 14.37	53.50 ± 14.35	81.76 ± 13.57	80.59 ± 14.76	85.42 ± 14.22	<0.05
Folate	29.64 ± 9.963	29.17 ± 7.012	35.00 ± 3.559	8.327 ± 2.756	8.342 ± 2.989	7.400 ± 1.776	4.241 ± 1.577	4.441 ± 1.691	3.667 ± 1.557	<0.05

The *p*-value was calculated by GraphPad prism (version 5.01) by using one-way ANOVA and Tukey’s Multiple Comparison test.

**Table 10:** Studies showing the association between MTHFR C677T polymorphism and myocardial infarction.

Country	Control	MI	Genotype Frequency Control			Genotype Frequency in MI			*p* Value	Ref.
			CC	CT	TT	CC	CT	TT		
Turkey	242	231	123	111	8	119	89	23	0.004	[46]
Italy	1210	1210	363	317	230	371	547	292	0.026	[47]
Japan	2291	1192	804	1134	353	375	579	247	0.152	[48]
Italy	909	1626	311	436	162	574	754	298	0.84	[49]
Sweden	971	514	489	392	90	265	205	44	0.37	[50]
India	120	100	41	10	1	17	3	0	0.451	[51]
Egypt	50	50	22	22	6	22	24	4	0.466	[25]
Turkey	129	107	72	47	10	66	34	7	0.369	[52]
Sweden	514	971	574	754	298	311	436	162	0.66	[53]
Egypt	31	20	14	16	1	3	15	0	0.07	[54]
Mexican	167	167	42	78	47	38	75	54	0.69	[15]
Turkey	70	70	46	16	8	50	12	8	0.58	[55]
China	231	406	88	95	48	80	172	154	0.007	[56]
Tunisia	207	310	123	78	6	131	121	58	0.039	[57]
Iran	150	150	88	55	7	69	74	7	0.66	[58]

## Data Availability

All data generated and/or analyzed during this study are included in this published article.
